# High-Yield Di-Rhamnolipid Production by *Pseudomonas aeruginosa* YM4 and its Potential Application in MEOR

**DOI:** 10.3390/molecules24071433

**Published:** 2019-04-11

**Authors:** Zhuangzhuang Li, Yumin Zhang, Junzhang Lin, Weidong Wang, Shuang Li

**Affiliations:** 1 College of Biotechnology and Pharmaceutical Engineering, Nanjing Tech University, Nanjing 210009, China; 970344745@njtech.edu.cn; 2 College of Food Science and Light Industry, Nanjing Tech University, Nanjing 210009, China; 201861118008@njtech.edu.cn; 3 Oil Production Research Institute, Shengli Oil Field Ltd. Co. Sinopec, Dongying 257000, China; linjunzhang.slyt@sinopec.com (J.L.); wangweidong168.slyt@sinopec.com (W.W.)

**Keywords:** rhamnolipid, di-rhamnolipid, biosurfactant, microbial enhanced oil recovery, *Pseudomonas aeruginosa*

## Abstract

Rhamnolipids are a mixture of the homologs species due to variations in the rhamnose units and β-hydroxy fatty acid moieties, mainly including Rha-C_10_-C_10_, Rha-Rha-C_10_-C_10_, and Rha-C_10_. In this study, strain *P. aeruginosa* YM4 was selected for its capacity to efficiently produce di-rhamnolipid (Rha-Rha-C_10_-C_10_) as the predominant component with soybean oil and glycerol as carbon source, accounting for 64.8% and 85.7% of total products, respectively. The critical micelle concentration (CMC) of rhamnolipid products varies with the content of di-rhamnolipid, whereby lower CMC values corresponding to higher di-rhamnolipid contents. The rhamnolipids containing 85.7% di-rhamnolipid had the lowest CMC value of 50 mg/L. Accordingly the viscosity-reducing efficiency and oil-washing efficiency of rhamnolipids increased with higher di-rhamnolipid component. At a concentration of 500 mg/L, the rhamnolipids containing 85.7% di-rhamnolipid worked best and showed 82.5% oil-washing efficiency, which offered great promise for applications in enhanced oil recovery. The results showed the variation of structure and composition of rhamnolipids had a significant effect on their application.

## 1. Introduction

Rhamnolipids, which belong to the broad biomolecule class of glycolipids, are the most intensively studied biosurfactants. As the name implies, rhamnolipids contain a hydrophilic group made up of one or two (l)-rhamnose units and a hydrophobic group consisting of one or two β-hydroxy fatty acid moieties. Rhamnolipid surfactants were shown to reduce the surface tension of water from 72 to 28 mN/m, and the interfacial tension of water–oil systems from 43 to <1 mN/m [1,2,3]. They display both prominent surface activity and high emulsifying activity, which make them well suited for application in microbial enhanced oil recovery (MEOR). Rhamnolipid-producing microorganisms are used for in situ treatment of oil-containing sands or in ex situ applications [4,5,6].

Rhamnolipids are primarily produced by *Pseudomonas aeruginosa* strains. Researchers have long focused on strain improvement and process optimization to acquire high-yielding rhamnolipid producers [7,8]. These rhamnolipids can be mixtures of four to 28 types, mainly including Rha-C_10_-C_10_, Rha-Rha-C_10_-C_10_, and Rha-C_10_ [9,10,11]. Among them, di-rhamnolipid exhibits special characteristics, such as a lower critical micelle concentration and better bioavailability [12,13,14,15].

Although the use of rhamnolipids in enhanced oil recovery has been tested before, until recently, the production of rhamnolipid variants and their efficiency in MEOR have become a research hotspot [16,17,18]. In this study, the rhamnolipid-producing strain *P. aeruginosa* YM4 was selected for its capacity to efficiently produce Rha-Rha-C_10_-C_10_ as the major component with soybean oil and glycerol as carbon source. The rhamnolipid products, homolog composition, and enhanced oil recovery properties, including surfactant activity, emulsifying capability, viscosity-reducing efficiency, and oil-washing efficiency, were tested in this work. The result of rhamnolipid homolog composition and their applicability to MEOR should contribute to the potential use of the rhamnolipid products and optimization of products composition. 

## 2. Results

### 2.1. Production of Rhamnolipids

Rhamnolipids were produced by *P. aeruginosa* YM4 and the parental strain in shake flask using glycerol and soybean oil as carbon sources for 96 h; the titer of rhamnolipid products of the parental strain was 16.7 g/L and 17.9 g/L, respectively; the titer of rhamnolipid products of YM4 was 23.9 and 25.1 g/L, respectively (Table 1). The rhamnolipids production by YM4 increased by 30%. Both glycerol and soybean oil could be used as carbon sources to efficiently produce rhamnolipid by YM4 with conversion yield of approximately 80 to 85%.

### 2.2. Proportions of Rhamnolipid Variants Produced by P. aeruginosa YM4 

**Figure 1:**
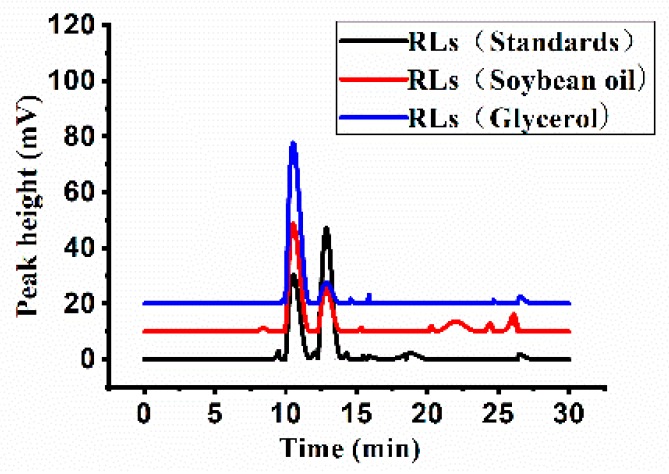
High performance liquid chromatography-evaporative light scattering detector (HPLC-ELSD) profile of rhamnolipids produced by *P. aeruginosa* YM4. The black line represents the rhamnolipid standard and the red and blue lines represent the rhamnolipids products from soybean oil and glycerol, respectively.

**Figure 2:**
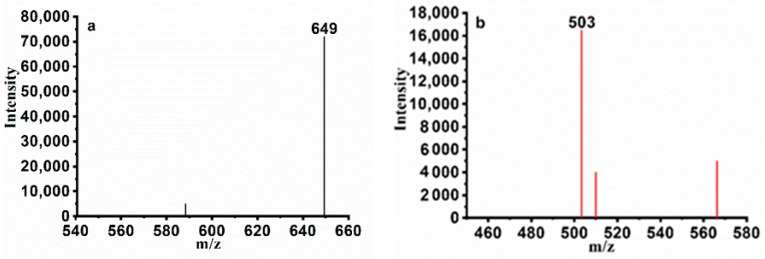
HPLC-MS analysis of the rhamnolipids. LC–MS spectrum of main components (retention times of 10.023 min (**a**) and 12.664min (**b**)) of the rhamnolipids.

HPLC profiles of the mixture of rhamnolipid variants produced by *P. aeruginosa* YM4 are shown in Figure 1. The rhamnolipid variants were identified by comparing their retention times and mass spectra with those of commercially available authentic reference standards. The main components appeared as peaks 1 and 2 with molecular masses of 649 (Rha-Rha-C_10_-C_10_) and 503 (Rha-C_10_-C_10_), respectively (Figure 2). 

The proportions of rhamnolipid products obtained with the two carbon sources were shown in Table 2. Among the rhamnolipid products of the strain YM4, Rha-Rha-C_10_-C_10_ and Rha-C_10_-C_10_ were the most abundant, accounting for approximately 90 to 95% of the total. However, the di-rhamnolipid proportion of the products was significantly affected by the carbon source, varying from 64.8 to 85.7%. When glycerol was used as carbon source, the contents of Rha-Rha-C_10_-C_10_ reached a maximum of 85.7%, and the ratio of di-rhamnolipids to mono-rhamnolipids was approximately 9:1. However, the mono-rhamnolipid proportion increased significantly with soybean oil as carbon source; and the ratio of di-rhamnolipids to mono-rhamnolipids was approximately 2:1. 

### 2.3. Measurement of the Critical Micelle Concentration (CMC)

**Figure 3:**
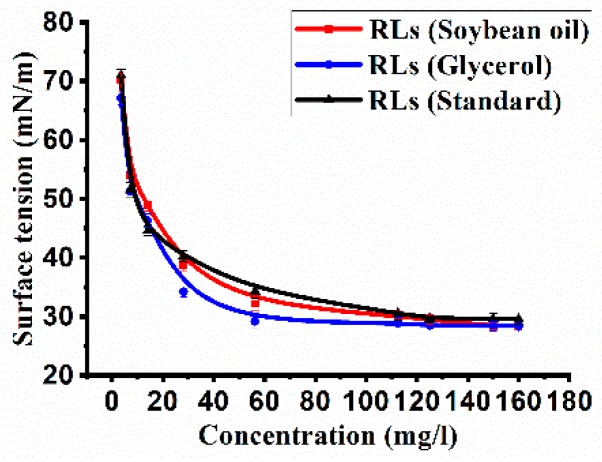
The critical micellar concentration (CMC) determination for rhamnolipids. The CMC was determined by measuring the changes of surface tension with different concentrations of rhamnolipids. The error bars represent standard deviations from three independent experiments (*n* = 3).

The rhamnolipids were able to reduce the surface tension of water from 72 to 28 mN/m at room temperature (25 °C). The relationship between the surface tension and the structure of rhamnolipids was analyzed by measuring the surface tension of rhamnolipid mixtures at different concentrations (0–200 mg/L; Figure 3). The CMC values of the rhamnolipid products from glycerol and soybean oil were 50 and 65 mg/L, respectively. By contrast, the CMC value of the standard sample (di-rhamnolipid accounting for 31.8%) was ~110 mg/L. The most significant difference between the rhamnolipid products and the standard sample was the proportion of di-rhamnolipid; the rhamnolipid products containing 85.7 and 64.8% of di-rhamnolipid, much higher than those in the standard sample (31.8%). The results showed the critical micelle concentration (CMC) of rhamnolipids was closely correlated with the composition of rhamnolipid variants, lower CMC values corresponding to higher di-rhamnolipid contents.

### 2.4. Emulsification Activity of Rhamnolipid Products

**Figure 4:**
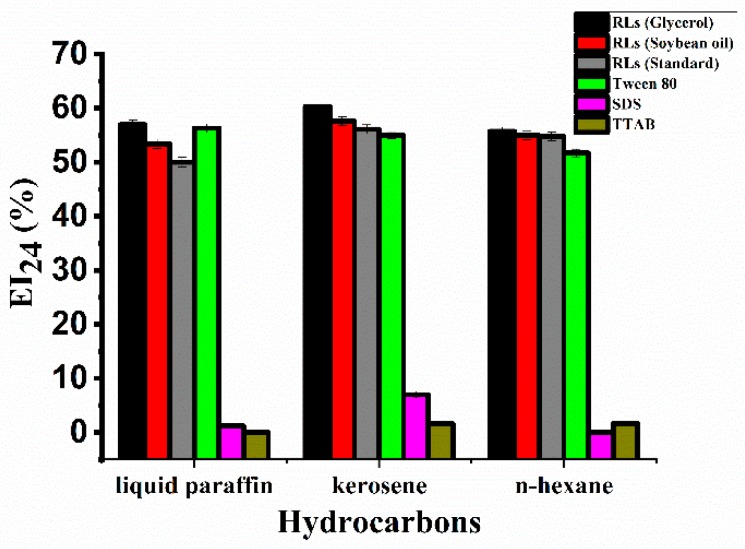
The emulsification index at 24 h (EI_24_) of surfactants in the different hydrocarbons. To measure EI_24_, 5 mL of surfactant solution was added to 5 mL of hydrocarbons in a glass test tube; the mixture was vortexed at high speed for 2 min, and incubated at room temperature for 24 h. The concentration of all surfactants was 500 mg/L. The error bars represent standard deviations from three independent experiments (*n* = 3).

In addition to surface tension, the stability of oil-in-water emulsions is often used as an indicator of surface activity [19]. The emulsification activity of rhamnolipid products, SDS, TTAB, and Tween 80 are shown in Figure 4. The rhamnolipid products were able to emulsify liquid paraffin, kerosene and n-hexane. Compared with SDS and TTAB, the rhamnolipid products and Tween 80 showed a more stable and efficient emulsification activity at a concentration of 500 mg/L. The emulsifying activity of different components of rhamnolipid products did not differ significantly.

### 2.5. Viscosity-Reducing Efficiency of Rhamnolipid Products 

Four crude oil samples were used to access the viscosity-reducing efficiency of rhamnolipid products at the same concentration. The results were shown in Table 3. The rhamnolipids (G) containing 85.7% di-rhamnolipid had a better viscosity-reducing effects than rhamnolipids (S) containing 64.8% di-rhamnolipid. This indicated that higher di-rhamnolipid content had better viscosity-reducing activity. 

### 2.6. Oil-Washing Efficiency of Rhamnolipid Products

**Figure 5:**
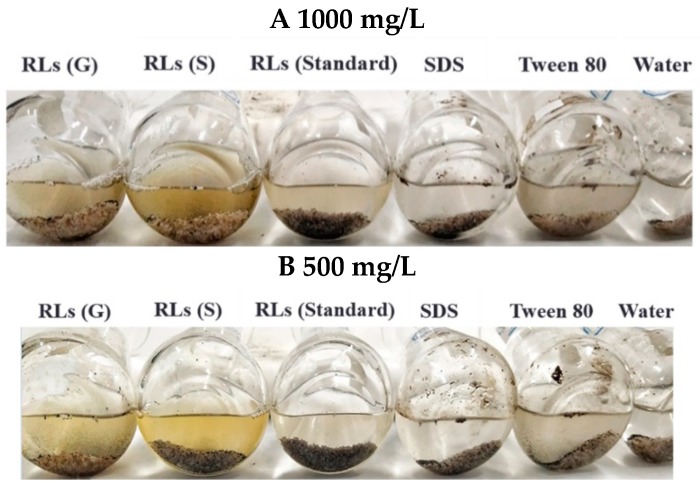
Bottom view of the oil sands after oil-washing by surfactants. The oil sands were treated with the surfactant solutions for 24 h at 70 °C and 90 rpm (**A**,**B**). RLs (glycerol) refer to the rhamnolipid products from glycerol; RLs (Soybean oil) refer to the rhamnolipid products from soybean oil. RLs (G) means rhamnolipids products from glycerol; RLs (S) means rhamnolipids products from soybean oil.

The performance of rhamnolipids products, rhamnolipid standards, as well as SDS and Tween 80 on oil-washing was studied using artificial oil sand. The results were shown in Table 4. At the concentration of 1000 mg/L, rhamnolipid (G) and Tween 80 removed more than 99% of oil from the oil sand, followed by rhamnolipid (soybean oil), which removed 87.5% of the oil. However, the rhamnolipid standard removed 51.7% of the oil, much less than the rhamnolipid products. At the concentration of 500 mg/L, rhamnolipid (glycerol) worked best: it could still remove more than 80% of the oil. The results suggested the components of rhamnolipids had a great influence on oil-washing efficiency, with a higher di-rhamnolipid content increasing the oil-washing efficiency. Although the chemical surfactant Tween 80 exhibited excellent oil-washing efficiency, it should be noted that the removed oil particles easily adhered to the wall of the flask (Figure 5). By contrast, the oil particles removed by the rhamnolipid products were homogeneously dispersed, which greatly simplifies the subsequent treatment.

## 3. Discussion

Rhamnolipids are mainly known to be produced by *Pseudomonas aeruginosa* during cultivation on specific substrates, the most commonly used being plant oils, sugars, and glycerol. Many studies have demonstrated that the fermentation conditions and carbon sources can dramatically affect the yield and composition of rhamnolipids. 

Carbon sources can affect the supply of precursors for rhamnolipid biosynthesis, thus *P. aeruginosa* strains are able to produce various rhamnolipid variants, which may differ in the chain length of the fatty acids or in the rhamnose units, when growing on different substrates [20]. While growing on glycerol and soybean oil, the ratios of Rha-Rha-C_10_-C_10_/Rha-C_10_-C_10_ in the products of *Pseudomonas aeruginosa* J16 were 4.2 and 2.3, respectively. After optimizing medium components for enhanced di-rhamnolipid production, the di-rhamnolipid accounted for 75 ± 5% of total rhamnolipid. However, the maximum concentration and volumetric productivity for di-rhamnolipid was 3.19 g/L and 44 mg/(L.h), respectively; the product concentration and volumetric productivity were relatively low and not suitable for industrial applications [21].

The biosynthesis of rhamnolipids needs two precursors—dTDP-L-rhamnose and beta-hydroxy fatty acids. The synthesis of Rha-C_10_-C_10_ is catalyzed by rhamnosyltransferase I (encoded by rhlAB) combining dTDP-L-rhamnose and β-hydroxydecanoyl-β-hydroxydecanoate, while Rha-Rha-C_10_-C_10_ is synthesized from Rha-C_10_-C_10_ and dTDP-L-rhamnose with the aid of rhamnosyltransferase II (encoded by rhlC). Tiso et al. designed and constructed a recombinant *Pseudomonas putida* KT2440 to specifically produce different biosurfactant mixtures via the tailored expression of rhamnolipid synthesis genes from *P. aeruginosa* PA01 in recombinant *Pseudomonas putida* cell factories [18]. First, the recombinant *Pseudomonas putida* could produce a mixture of different mono-rhamnolipid congeners, and then, after transforming the rhlC containing vector, the new recombinant was able to produce di-rhamnolipids. Of the total biosurfactants, 86% were di-rhamnolipids and 13% were mono-rhamnolipids. This was the first report of efficient di-rhamnolipids production in recombinant bacterium. It should be noted the function genes donor *P. aeruginosa* PA01 also produced mainly the Rha-Rha-C_10_-C_10_ congener; The molar ratio of di-rhamnolipid to mono-rhamnolipid in the products of *P. aeruginosa* PA01 with sunflower oil was approximately 2:1 [22].

In this study, the rhamnolipid yields were not significantly affected by the type of carbon source; both glycerol and soybean oil could be efficiently used by the YM4 strain with conversion yields of 80–85% (Table 2). However, the composition of rhamnolipids differed significantly, and the di-rhamnolipid proportion varied from 64.8 to 85.7%. These yields and proportions make *Pseudomonas aeruginosa* YM4 an unusually efficient di-rhamnolipid (Rha-Rha-C_10_-C_10_) producer. Especially when using glycerol as carbon source, the rhamnolipid products contained a di-rhamnolipid proportion as high as 85.7%. Hanser et al. showed that the rhamnosyl moiety of rhamnolipids can be directly condensed from two glycerol molecules that are not rearranged in their carbon chains, and the fatty acid chain portion can also be obtained by a fatty acid synthesis route from glycerol [23]. Therefore, glycerol has a metabolic advantage for the glycosylation of the rhamnolipid and the synthesis of the lipid-based moiety. This may also be the reason why YM4 utilizes glycerol to produce di-rhamnolipids at a higher yield. A comparison of the reported rhamnolipid variants produced by different strains is shown in Table 5. To the best of our knowledge, this is the highest yield of di-rhamnolipid produced by *Pseudomonas* species.

Rhamnolipid congeners with variable numbers of hydrophobic/hydrophilic residues and their mixtures feature different physicochemical properties that might lead to diverse applications. The composition and distribution of homologs greatly affect the surface activity characteristics of rhamnolipid preparations. Marcia et al. pointed out that rhamnolipids with longer fatty acid chains had stronger hydrophobicity, but they were more hydrophilic if the sugar component was di-rhamnose [28]. Therefore, the variation of structure and composition can affect the surface activity and stability of rhamnolipids in the aqueous phase [21]. The critical micelle concentration (CMC) of rhamnolipid is also affected by the length of the fatty acid chain and the number of rhamnose units. If the rhamnolipid structure contains two rhamnoses, a longer fatty acid chain leads to a lower CMC [12,20]. Lang et al. reported that the di-rhamnolipid Rha-Rha-C_10_-C_10_ has a CMC of 5 mg/L, which is much lower than that of Rha-C_10_-C_10_, which has a value of 40 mg/L [29]. Among the rhamnolipid products of the YM4 strain, the main congeners were Rha-Rha-C_10_-C_10_ and Rha-C_10_-C_10_, accounting for approximately 90–95% of the total. Rhamnolipid products with a higher percentage of di-rhamnolipid (Rha-Rha-C_10_-C_10_) had lower CMC values, and both of the products showed much lower CMC values than the commercially available rhamnolipid standards (Figure 3). Gong et al. studied the influence of different congeners of rhamnolipids on interfacial tension (IFT), and found that a high percentage of di-rhamnolipid (Rha-Rha-C_10_-C_10_) facilitates a decrease of the IFT [24]. Considering the lower CMC value and IFT of di-rhamnolipid, it is easy to explain the better oil-washing efficiency and viscosity reduction that was observed in the rhamnolipid product containing 85.7% of di-rhamnolipid. The present results suggest the di-rhamnolipids would be the better choice in the MEOR application.

To be competitive with chemical surfactants, many works focused on enhancing rhamnolipid titers and yields by selecting suitable substrates and optimizing the fermentation process. However, the contents of biosurfactant isoforms or congers also have a significant effect on their application. The lower CMC values and better oil-washing efficiency of di-rhamnolipid observed in this study indicates that the proportion of di-rhamnolipid should be paid special attention in rhamnolipids production for MEOR applications.

## 4. Materials and Methods

### 4.1. Bacterial Strain and Culture Conditions

*P. aeruginosa* YM4 (CCTCC No.: M2017494), was originally isolated from crude oil contaminated soil samples and obtained via random mutagenesis with atmospheric room temperature plasma (ARTP). The strain was cultured in Luria-Bertani (LB) medium (10 g/L peptone, 5 g/L yeast extract, 10 g/L NaCl) at 200 rpm and 37 °C for 12 h. The optic density of seed culture was about 2.0–2.5, and it was then transferred into the 250 mL flask containing 50 mL fermentation medium at a 3% (*v*/*v*) inoculation rate. The fermentation for rhamnolipid production was continued at 37 °C and 200rpm for 96 h. The optimal fermentation medium comprised (g/L) K_2_HPO4·3H_2_O (4), KH_2_PO_4_ (4), MgSO_4_·7H_2_O (0.2), CaCl_2_ (0.1), yeast extract (1), trace elements solution (2.5 mL/L), and carbon and nitrogen sources comprising glycerol (30), NaNO_3_ (12), soybean oil (30), or NaNO_3_ (8), respectively. The trace elements solution contained (g/L) FeCl_3_ (0.16), CuSO_4_ (0.15), ZnSO_4_·7H_2_O (1.5), and MnSO_4_·H_2_O (1.5).

### 4.2. Extraction and Purification of Rhamnolipids

Cells were separated from 100 mL of fermentation broth by centrifugation (10,956 × g, 15 min, 20 °C, Hitachi CR21GIII, Tokyo, Japan). The supernatant was subjected to acid precipitation by the addition of 10 M HCl to give a final pH of 2.0 and the precipitate was formed overnight at 4 °C. The precipitate was harvested by centrifugation (10,956× *g*, 15 min, 4 °C) and extracted with 300 mL ethyl acetate. The organic phase was collected, and the solvent was removed in a rotary evaporator (Hei-VAP Industrial, Berlin, Germany) leaving a brown paste. The paste was re-extracted with 300 mL ethyl acetate, and the supernatant was collected by centrifugation (10,956× *g*, 15 min, 4 °C). Finally, the crude rhamnolipids were obtained by rotary evaporation.

### 4.3. HPLC-ELSD Analysis

Rhamnolipids were detected directly, without derivatization, by HPLC using an Evaporative Light Scattering Detector (ELSD), as described previously [30]. 

After the end of the fermentation, 0.1 mL of the cell-free supernatant was mixed with 1.9 mL of absolute ethanol and shaken for 1 min, and then separated by centrifugation (10,956× *g*, 5 min, 4 °C). The supernatant was analyzed using a 1260 Infinity HPLC-ELSD (Agilent Technologies, Palo Alto, CA, USA) equipped with a C18 column (4.6 × 150 mm, 5 μm; Sepax Technologies, Inc, Suzhou, China). The ELSD was set up with a drift temperature of 60 °C and a nebulizer flow rate of 1.5 L/min. The mobile phase A (acetonitrile) and mobile phase B (water with 0.05% formic acid) were used in a linear gradient from 30% to 100% of acetonitrile in 40 min. Standard curves comprising 0.1 to 1 mg/mL of rhamnolipids (AGA102317b; AGAE Technologies, Corvallis, OR, USA) were used for quantification.

### 4.4. HPLC-MS Analysis

Sample pretreatment was the same as Section 4.3. For quadrupole-time of flight (Q-TOF ) analysis, the analytes were separated as described above, and mass spectrometry was conducted as follows; the nebulized gas and dry gas were N_2_, the ESI source was used in the high performance liquid chromatography-quadrupole-time of flight-mass spectrometry (HPLC/Q-TOF-MS) system: ion source temperature was 350 °C, ion source gas flow rate was 10 L/min, spray voltage was 45P, and the mass spectrum scanning range was 200 to 1000 *m*/*z*.

### 4.5. Determination of the Critical Micelle Concentration (CMC) of Rhamnolipids

The critical micelle concentration (CMC) was determined by measuring the surface tension curve of the separated biosurfactant dilution as described before [31]. The changes in surface tension of rhamnolipid solutions with different concentrations (0–200 mg/L) were measured using an automated tensiometer (BZY-3B, Shanghai Automation Instrumentation Sales Center, Shanghai, China) at room temperature.

### 4.6. Determination of the Emulsification Index (EI_24_)

The emulsification index (EI_24_) was measured using the method described by Cooper and Goldenberg [32]. Briefly, 5 mL solutions of different chemical surfactants (Sodium dodecyl sulfate (SDS), Tetradecyl trimethyl ammonium Bromide (TTAB), Tween 80) and rhamnolipid products, and 5 mL of different hydrocarbons (n-hexane, liquid paraffin, kerosene) were mixed in 10 mL stoppered glass test tubes, in different combinations. The mixtures were vortexed at high speed (~10,000 rpm) for 2 min, and subsequently kept without agitation at room temperature for 24 h. The EI_24_ was calculated by dividing the measured emulsion layer height by the total height of the mixture and multiplying by 100.

### 4.7. Assessment of the Viscosity-Reducing Effect of Rhamnolipids on Crude Oil

To investigate the effect of rhamnolipids on the viscosity crude oil, 30 g of rhamnolipids solution (5 g/L) and 70 g of crude oil were mixed in a glass beaker, and viscosity of the mixtures was measured immediately after heating at 50 °C for 2 h using viscometer (Shanghai Biaozhuo Scientific Instrument Co., Ltd., Shanghai, China). Then, the mixtures were kept at room temperature overnight and the viscosity was measured again. The crude oil samples used in the experiment were supplied by the Shengli Oilfield Microbial Oil Recovery Research Institute, Dongying, China. 

### 4.8. Assessment of the Oil-Washing Efficiency of Rhamnolipids on Oil Sands 

Standard oil sand for measuring the oil washing efficiency was obtained by mixing 170 g of quartz sand (Shengli Oilfield, China), 4 g of man-made crude oil (83% crude oil, 12% petroleum asphalt, 5% paraffin (m/m); Shengli Oilfield, Dongying, China), and 10 mL petroleum ether (China Pharmaceutical Chemical Reagent Co., Ltd., Beijing, China) with stirring and heating at 80 °C for 1 h, and then evaporating the petroleum ether at 80–90 °C. Sequel samples containing 20 mL of surfactant solution were added to each flask containing 2 g of the thus-formed oil sand. The flask was then shaken at 90 rpm and 70 °C for 24 h. The oil removed from the solution was transferred to a new container, and the remaining detergent solution was rinsed in a flask with distilled water until the eluent was clear. The washed flask with oil sand was then placed into the oven and dried at 60 °C for 12 h, cooled to room temperature in a desiccator, and the dry weight of the oil sand measured. The oil-washing efficiency was calculated using the formula:

(1) $$X=\frac{W0-W1}{W0*k}\times 100\%$$

*X*—Oil-washing efficiency, *%*;
*W*0—Added oil sands quality, g;
*W*1—Dried oil sands quality, g;
*k*—The proportion of oil in oil sands, *%*.

## 5. Conclusions

Strain *P. aeruginosa* YM4 was able to efficiently produce di-rhamnolipid (Rha-Rha-C_10_-C_10_) as the predominant component with soybean oil and glycerol as carbon source, accounting for 64.8% and 85.7% of total products, respectively. The results of enhanced oil recovery properties, including surfactant activity, emulsifying capability, viscosity-reducing efficiency, and oil-washing efficiency, of the two types of rhamnolipid products indicated the proportion of di-rhamnolipid in rhamnolipids production was one of the key factors for MEOR applications.

## Figures and Tables

**Table 1:** Effects of the carbon source on rhamnolipid production.

Strain	Carbon Source	Biomass (OD_600_)	Productivity (mg/L/h)	Titer (g/L)	Conversion Yield
YM4	glycerol	5.1 ± 0.2	249.0 ± 12.5	23.9 ± 1.2	79.7 ± 4.0%
	soybean oil	7.0 ± 0.2	260.9 ± 14.6	25.1 ± 1.4	83.5 ± 4.6%
Parent	glycerol	5.8 ± 0.3	173.9 ± 24.0	16.7 ± 2.3	55.7 ± 7.6%
	soybean oil	7.4 ± 0.2	186.4 ± 16.7	17.9 ± 1.6	59.7 ± 5.3%

**Table 2:** The components of rhamnolipid products.

Structure	Retention Time (min)	Rhamnolipid Preparations		
		Glycerol	Soybean Oil	Standard
Rha-Rha-C_10_-C_10_	10.023	85.7%	64.8%	31.8%
Rha-C_10_-C_10_	12.664	9.3%	27.0%	64.0%
Rha-C_12:1_	15.416	3.0%	ND	ND
Rha-C_14:2_-C_10_	18.536	ND	ND	1.9%
Rha-C_10:1_	20.157	ND	5.2%	ND
Rha-C_10:1_-C_8_	22.117	ND	3.0%	ND
Rha-C_14:1_	25.987	2.0%	ND	2.3%

ND: not detectable.

**Table 3:** Viscosity-reducing effect of rhamnolipid products.

Crude Oil	Viscosity (mPas)	Viscosity-Reducing (2h/Overnight)	
		RLs (G) ^a^	RLs (S) ^b^
CQ63	585	95.7%/91.5%	79.4%/76.3%
33	3654	99.2%/14.5%	89.2%/7.7%
68-73	7948	96.2%/18.4%	84.6%/15.2%
ZEN	14187	99.9%/32.6%	99.1%/19.9%

Thirty grams of rhamnolipid solution (5 g/L) was added to 70 g of crude oil, incubated at 50 ° C for 2 h, and then stirred evenly to determine the viscosity of mixture. After staying still at room temperature overnight, the viscosity of mixture was determined again. Each batch of experiment was repeated 3 times. ^a^ RLs (G) means rhamnolipids products from glycerol; ^b^ RLs (S) means rhamnolipids products from soybean oil.

**Table 4:** Oil-washing efficiency of surfactant solutions.

Surfactant	Concentration (mg/L)	% of Oil Removed
RLs (Glycerol) 85.7% di-rhamnolipids	1000	99.3 ± 0.3
	500	82.5 ± 1.1
RLs (Soybean oil) 64.8% di-rhamnolipids	1000	87.5 ± 2.0
	500	53.7 ± 1.7
RLs (Standard) 31.8% di-rhamnolipids	1000	57.1 ± 1.4
	500	28.8 ± 2.2
SDS	1000	38.7 ± 0.9
	500	25.0 ± 1.3
Tween 80	1000	99.6 ± 0.1
	500	87.2 ± 1.5
Water	-	37.7 ± 1.6

A sample comprising 2 g of artificial oil sand was added to 20 mL of surfactant solutions in 50 mL flasks, and then shaken at 90 rpm and 70 °C for 24 h. The error bars represent standard deviations from three independent experiments.

**Table 5:** The rhamnolipid products and di-rhamnolipid contents from various strains.

Strain	Carbon Source	Conversion Yield (%)	Content of Di-Rhamnolipid (%)	Surface Tension (mN/m)	Reference
*P. aeruginosa* J16	Glycerol	9	75	NA	[21]
*P. aeruginosa* TIB-R02	Palm oil	80	60	NA	[24]
*P. aeruginosa* 57RP	Mannitol	NA	~55–60	NA	[20]
*Recombinant P. putida*	Glucose	33	86	NA	[18]
*P. aeruginosa* L2-1	Waste oil	NA	~70–75	30	[25]
*P. aeruginosa* MR01	Soybean oil	NA	42.7	29.9	[3]
	Glucose	24	77.2	NA	[26]
*P. aeruginosa*E03-40	Glycerol	10	64	NA	[27]
	Soybean oil	47	18	NA	
*P. aeruginosa*YM4	Glycerol	79.7	85.7	29.2	This study
	Soybean oil	83.5	64.8	32.2	

NA: not available.
